# Hemolytic anemia after mitral valve repair: a case report

**DOI:** 10.1186/1756-0500-6-165

**Published:** 2013-04-25

**Authors:** Catarina Cardoso, Patricia Cachado, Teresa Garcia

**Affiliations:** 1 Department of Internal Medicine, Santa Marta Hospital, Lisbon, Portugal

**Keywords:** Hemolytic anemia, Mitral valve repair, Regurgitant jet, Heart failure

## Abstract

**Background:**

Hemolytic anemia after mitral valve repair is still an underestimated complication because it is a rare condition and there are few described case reports in the literature. The mechanism responsible for hemolysis most commonly involves a regurgitant jet and it appears to be independent of its severity as assessed by echocardiography. Patients may experience severe symptoms with only moderate regurgitant jets.

**Case presentation:**

We present a case of a 74-year-old Caucasian female who developed severe hemolytic anemia and decompensated heart failure due to a moderate mitral valve regurgitation jet, but it was not considered severe enough to explain such hemolysis. After exclusion of other causes of hemolytic anemia and the lack of clinical and laboratory improvement, the patient underwent valve replacement with a mechanical valve. Anemia and heart failure symptoms gradually resolved after surgery.

**Conclusion:**

This case report indicates the importance of including the diagnosis of hemolytic anemia after mitral valve repair in patients with severe hemolysis and a surgical history of heart surgery, even if echocardiography underestimates or is unclear for showing significant alterations. The interest on mitral valve repair as suggested by the increase in the number of these procedures performed worldwide, raises the possibility that hemolytic anemia could be probably seen more frequently in the future and be a cause of major concern, especially for cardiac surgeons.

## Background

A certain degree of hemolysis after heart valve surgery is relatively common, although it may progress to severe forms with hemolytic anemia and require transfusion support [1]. In these cases, valve structure deterioration or paravalvular leakage usually occurs [2]. In rare situations, hemolytic anemia can also occur in normal-functioning prosthetic valves or even in native valves. Mechanical trauma is the main cause of hemolysis and the mechanism most frequently associated with hemolysis is turbulence of outflow caused by a valve’s configuration and/or functioning [1]. With an increasing number of valve repairs, hemolytic anemia has been reported only in a few cases [3-6].

We present a case of severe hemolytic anemia, which was initially in doubt as an exclusive complication of mitral valve repair, not only because it is rare, but also because of echocardiographic findings. We also describe the pharmacological treatment used to compensate for the patient’s symptoms and review the literature regarding this condition.

### Case presentation

**Figure 1:**
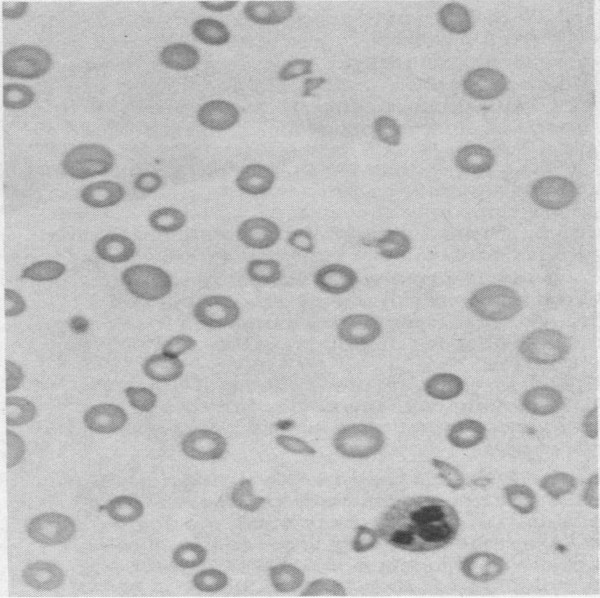
Patient’s peripheral blood smear showing fragmented red blood cells suggesting schistocytes.

A 74-year-old female with known arterial hypertension, chronic atrial fibrillation, dyslipidemia, and severe mitral regurgitation with asymptomatic severe pulmonary hypertension was diagnosed by routine echocardiography. Mitral valve repair was performed in October 2010 without apparent complications. She was discharged at day 6 post-surgery and had already complained about shortness of breath and bilateral leg edema. At day 18 post-surgery, she went to the emergency department because of worsening symptoms and she also complained of dark urine, jaundice, and right abdominal pain. A physical examination revealed extensive jaundice, with jugular ingurgitation at 45°, shortness of breath at rest, auscultation with systolic murmur audible in the precordium, hepatomegaly, and bilateral leg edema. Laboratory values showed normocytic and normochromic anemia of 7 g/L, elevated levels of transaminase, gamma-glutamyl transferase, and alkaline phosphatase, hyperbilirubinemia (18 mg/dL) with a direct fraction of 8,18, an elevated lactate dehydrogenase level of 4467 IU/L, low haptoglobin, hemoglobinuria, and the presence of schistocytes in a blood smear (Figure 1) (Table 1). A thoracic X-ray revealed cardiomegaly with pulmonary edema. Abdominal ultrasound showed biliary sludge.

**Figure 2:**
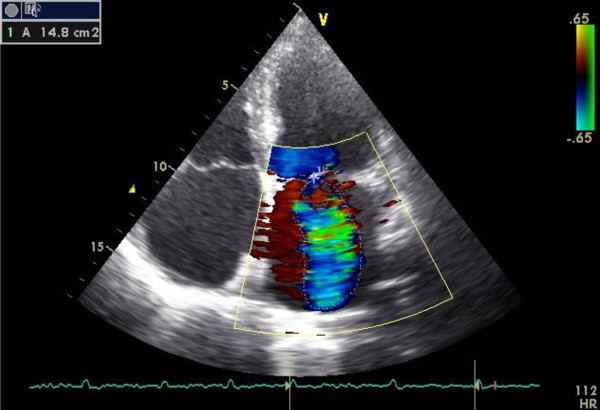
Moderate to severe mitral regurgitation (red) seen in echocardiography (parasternal view).

**Figure 3:**
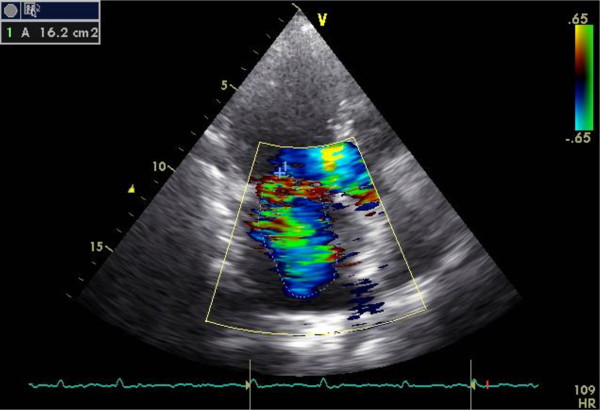
Mitral plasty regurgitation.

With the clinical picture of hemolytic anemia with right heart failure and a previous heart surgery, the patient had transthoracic echocardiography performed (Figure 2 and 3). We found mitral plasty with moderate mitral regurgitation (Figure 2), which was unlikely to be the cause of such severe hemolysis. The case was discussed with cardiac surgeons, who excluded the possibility of hemolytic anemia because of problems with mitral valve repair and advised investigation of other etiologies that might explain the patient’s condition.

**Table 1:** Laboratory findings at first admission to the emergency department

**Hemoglobin**	**7,0 g/L (12–15 g/L)**
Mean corpuscular volume	**88 (78–96 fL)**
Mean corpuscular hemoglobin	**30 (27–34 pg)**
Reticulocytes	**10% (0,5-1,5%)**
Alanine transaminase	**369 U/L (5–38 U/L)**
Aspartate transaminase	**890 U/L (6–34 U/L)**
Gammaglutamyl transferase	**200 U/L (5–55 U/L)**
Alkaline phosphatase	**224 U/L (20–140 U/L)**
Total bilirubin	**18 mg/dL (0,1-1,2 mg/dL)**
Direct bilirubin	**8,18 mg/dL**
Lactate dehydrogenase	**4467 U/L (0–250 U/L)**
Pro-BNP	**7440 pg/mL (0–99 pg/mL)**
Haptoglobin	**<0,01**

The patient was admitted in an internal medicine ward where she began intravenous diuretic therapy, transfusion support, iron, and folate, as well as an angiotensin-conversion enzyme blocker. Other hemolytic anemia causes were excluded.

She was discharged after symptomatic and blood values improved (Table 2), with a scheduled cardiothoracic appointment and transthoracic echocardiography.

**Table 2:** Improvement of the patient after first discharge

**Hemoglobin**	**9,3 g/L (12-15 g/L)**
Mean corpuscular volume	**88 (78–96 fL)**
Mean corpuscular hemoglobin	**30 (27–34 pg)**
Reticulocytes	**8% (0,5-1,5%)**
Alanine transaminase	**44 U/L (5–38 U/L)**
Aspartate transaminase	**75 U/L (6–34 U/L)**
Lactate dehydrogenase	**1710 U/L (0–250 U/L)**
Alkaline phosphatase	**121 U/L (20–140 U/L)**
Total bilirubin	**4,4 mg/dL (0,1-1,2 mg/dL)**

Despite her improvement, she began having the same complaints of shortness of breath and generalized edema, as well as abdominal discomfort, which resulted in her being admitted to the emergency department again. She had an Abdominal Computer Tomography Scan performed because of her abdominal complaints and hyperamylasemia of 1123 U/L. There was no evidence of acute pancreatitis and the patient was discharged with the same diagnosis of hemolytic anemia after valve repair. One week later, there was no further improvement, and she was re-admitted to our ward. Transthoracic echocardiography was similar to the first image, and despite the same pharmacological measures, she did not improve. She underwent another surgical procedure with mitral valve replacement. According to the cardiac surgeon, there was no macroscopic evidence of dysfunction in the previous mitral valve repair.

In the post-surgery period, the patient showed significant improvement of heart failure complaints and anemia (Table 3). Currently, the patient is asymptomatic for activity that requires a small amount of effort, and is anicteric, without any evidence of anemia (Table 4).

**Table 3:** Recovery from anemia 1 week after valve replacement surgery

**Hemoglobin**	**10,2 g/L (12–15 g/L)**
Mean corpuscular volume	**88 (78–96 fL)**
Mean corpuscular hemoglobin	**30 (27–34 pg)**
Reticulocytes	**0,5% (0,5-1,5%)**
Alanine transaminase	**75 U/L (5–38 U/L)**
Aspartate transaminase	**80 U/L (6–34 U/L)**
Lactate dehydrogenase	**340 U/L (0–250 U/L)**
Alkaline phosphatase	**120 U/L (20–140 U/L)**
Total bilirubin	**2,0 mg/dL (0,1-1,2 mg/dL)**

**Table 4:** Recovery from anemia 3 months after valve replacement

**Hemoglobin**	**14,2 g/L (12–15 g/L)**
Mean corpuscular volume	**88 (78–96 fL)**
Mean corpuscular hemoglobin	**30 (27–34 pg)**
Reticulocytes	**0,1% (0,5-1,5%)**
Alanine transaminase	**44 U/L (5–38 U/L)**
Aspartate transaminase	**47 U/L (6–34 U/L)**
Lactate dehydrogenase	**247 U/L (0–250 U/L)**
Alkaline phosphatase	**128 U/L (20–140 U/L)**
Total bilirubin	**0,8 mg/dL (0,1-1,2 mg/dL)**

## Discussion

Our skepticism for accepting the original diagnosis of our patient was mainly because of the degree of the anemia and the lack of echocardiographic findings that would exclusively explain such severe anemia. The clinical presentation of anemia can range from a mild to a severe form. It is well known that not all patients develop hemolytic anemia, presenting with only subclinical hemolysis with small increases in lactate dehydrogenase levels and low haptoglobin levels [2]. Previous studies have concluded that after mitral valve repair, minimal regurgitation is often undetected by intraoperative ultrasound [7]. Clinical evidence of hemolytic anemia usually manifests 90 days after surgery [7], but it may appear sooner, depending on the presence of certain factors that contribute to hemolysis; for example, for extracorporeal circulation through cardiopulmonary bypass, 12% hemolysis is estimated after 2 h on this mechanical system [8].

Mitral valve repair is quite effective in many cases and may be associated with a lower long-term morbidity and mortality than mitral valve replacement. The repair procedure includes shortening of tendinous chords, prolapsed leaflet resection, and annuloplasty [6]. Hypothesized hemolysis mechanisms include regurgitant jets in dehisced annuloplasty rings, red blood cell collision on perivalvular sutures, and residual tendinous chords [5]. These mechanisms delay endothelialization, maintaining hemolysis. Some authors believe that interindividual red blood cell fragility might play a part in hemolysis, as well as an auto-immune phenomenon, as indicated by some reports of successful treatment with corticosteroids [9].

Echocardiographic study in a case such as our patient is an important tool because it allows investigation of the regurgitant jet, as well as its origin. Echocardiography, especially transesophageal echocardiography in mitral regurgitation, is a sensitive and specific method [10].Small and asymptomatic paraprosthetic jets are often detected as a result of the high sensitivity of colour flow mapping.

The speed, direction, and impact location should be considered in the regurgitant jet. Distinct patterns of mitral regurgitant flow disturbances have been identified in patients with mitral prosthetic hemolysis. These include fragmentation, collision, acceleration, deceleration, and a free jet [7].

There are few pharmacological treatments to relieve anemia symptoms. Beta-blockers, pentoxifylline, intravenous diuretics, and iron supplements might improve symptoms [1,2]; however, they only alleviate symptoms temporarily.

The decision to reintervene (i.e., a second surgery) is a difficult one, in part because of the conflicting literature concerning the significance of mild-moderate residual regurgitation. Fix et al. found no increase in long-term mortality in patients with mild regurgitation after repair, although a trend for an increased late reoperation was noted [11]. In contrast, Sheikh et al. found an increase in postoperative morbidity and mortality in patients with mild to moderate residual regurgitation [12].

The condition of the heart, the potential for injury during a second cross-clamp period, and the potential need for valve replacement with its attendant costs should also be considered. These decisions require echocardiographers with a firm and confident grasp of echocardiography interpretation, surgeons with an awareness of their capabilities, and a clear understanding by the entire team of the short-term implications of reintervention and the long-term implications of residual mitral regurgitation.

## Conclusion

The purpose of this article was to describe a case of a rare cause of hemolytic anemia, as well as to highlight valve repair as a possible cause of hemolytic anemia. With an increasing number of this type of procedure, significant intravascular hemolysis is a cause of major concern, not only for cardiac surgeons, but also for cardiologists and internal medicine professionals, even when the repair is considered as adequate.

## Consent

Written informed consent was obtained from the patient for publication of this case report and accompanying images. A copy of the written consent is available for review by the Editor-in-Chief of this journal.

## Competing interests

The authors declare that they have no competing interests.

## Authors’ contributions

CC analyzed and interpreted the data of our patient, and was a major contributor in writing the manuscript. PC contributed to the revision and the writing of the manuscript. TG analyzed and interpreted the data of our patient. All authors read and approved the final manuscript.

## References

[B1] Rajiv M Larry J Alfred I Morris K Evaluation of hemolysis in patients with prosthetic heart valves Clin Cardiol 1998 21 387 392 10.1002/clc.4960210604 9631266 PMC6656177

[B2] Fatih K Lufti B Hasan K Koksal C Hemolysis and infective endocarditis in a mitral prosthetic valve Türk Kardiyol Dern Arş - Arch Turk Soc Cardiol 2010 38 6 429 431 21200125

[B3] Mestres C Intravascular hemolysis after mitral valve repair: a word of caution Eur J Cardiothoracic Surg 1992 6 103 105 10.1016/1010-7940(92)90084-B 1581078

[B4] Yasushige SMD Hidetoshi AMD Noriyoshi EMD Kazuhiro EMD Ko TMD Juriche OMD Takashi FMD A surgical case for severe hemolytic anemia after mitral valve repair Ann Thorac Cardiovasc Surg 2005 11 198 200 16030481

[B5] Inoue M Kaku B Kanaya H Ohka T Ueda M Masahiro S Shimizu M Mabuchi H Reduction of hemolysis without reoperation following mitral valve repair Circ J 2003 67 799 801 10.1253/circj.67.799 12939559

[B6] Ui T Misawa Y Oki S Saito T Fuse K Hemolytic anemia caused by mild regurgitation after mitral valve plasty Jichi Medical School Journal 2003 26 61 65

[B7] Tiong Y William F Hartzell S Thomas O Mechanisms of hemolysis after mitral valve repair: assessment by serial echocardiography J Am Coll Cardiol 1998 32 717 723 10.1016/S0735-1097(98)00294-0 9741517

[B8] Solberg R Extracorporeal circulation – effect of long term (24 hour) circulation on blood components PhD thesis. Faculty of the Virginia Polytechnic Institute, Science in Biomedical and Veterinary 201023644614

[B9] O’Regan S Newman AJ Cardiac hemolytic anemia resolving after second mitral valvuloplasty Can Med Assoc J 1976 115 419 420 953916 PMC1878713

[B10] Ionescu A Fraser A Butchart E Prevalence and clinical significance of incidental paraprosthetic valvar regurgitation: a prospective study using transoesophageal echocardiography Heart 2003 89 1316 1321 10.1136/heart.89.11.1316 14594888 PMC1767938

[B11] Fix J Isada L Cosgrove D Miller DP Savage R Blum J Stewart W Do patients with less than “echo-perfect” results from mitral valve repair by intraoperative echocardiography have a different outcome? Circulation 1993 88 II39 II48 8222184

[B12] Sheikh K DeBruijn N Rankin JS Clements FM Stanley T Wolfe WG Kisslo J The utility of transesophageal echocardiography and Doppler color flow imaging in patients undergoing cardiac valve surgery J Am Coll Cardiol 1990 15 363 372 10.1016/S0735-1097(10)80064-6 2299078

